# The Spanish General Knowledge Norms

**DOI:** 10.3389/fpsyg.2016.01888

**Published:** 2016-12-01

**Authors:** Jon A. Duñabeitia, Kim L. Griffin, Juan L. Martín, Mireia Oliva, María L. Sámano, Lela Ivaz

**Affiliations:** ^1^Basque Center on Cognition, Brain and LanguageDonostia, Spain; ^2^Facultad de Ciencias Sociales y Humanidades, Universidad Europea del AtlánticoSantander, Spain; ^3^Centro de Investigación y Tecnología Industrial de CantabriaSantander, Spain

**Keywords:** general knowledge, Spanish norms, cross-language comparison, cultural norms, memory recall, false belief

## Introduction

One of the building blocks of culture is general knowledge—culturally valued and cross-generational knowledge about historical facts. Possession of an elementary level of this knowledge is regarded as indispensable and has even become a key part of many naturalization tests. It is broadly assumed that the citizens of many countries worldwide should know the answers to a number of civics questions. Astonishingly, knowing the answers to questions such as “Who was the first U.S. President?” is viewed as an indicator of how well someone could integrate into a country. This speaks volumes to the importance of general knowledge. However, the facts that are deemed general knowledge depend to a great extent on the country, since the study of civics and culture is nearly always specific to a particular territory. As a result, there are no universal sets of cross-cultural general knowledge norms in academia. This is an impediment that further highlights the need to constantly update and validate these norms across different languages and countries. In the present paper, we attempt to bridge this gap by adapting U.S.-centric general norms to a Spanish-speaking population and testing them with a large sample of college students in Spain.

For over three decades, the most commonly-used set of cultural norms in psychological research has been the one published by Nelson and Narens (1980). This set includes 300 U.S.-centric, general-information questions of a fairly heterogeneous origin and different degrees of complexity, which were answered by 270 college students from the Universities of California and Washington. These norms have been extensively used in areas of research focusing on memory-related processes, constituting the largest normative pool of general-knowledge cultural questions (see Nelson et al., 1982; Marsh et al., 2003, 2005; Fazio and Marsh, 2008; Weinstein and Roediger, 2010).

Nelson and Narens (1980) meticulously selected “timeless” topics to avoid dramatic changes in the ease of recall over the course of years. However, their results were not impervious to aging. On that point, Tauber et al. (2013) realized that a three-decade gap was enough to shift people's general knowledge and to partially invalidate preceding results. After correcting some errors that were present in the original norms, Tauber et al. (2013) recruited a large number of participants and collected new norms to validate and update the materials. Interestingly, they extended the data collection to other relevant pieces of information such as confidence judgment (i.e., the percentage of likelihood to provide a correct response), and more importantly, commission errors (i.e., the most frequently reported incorrect responses for each question). This last addition yielded highly relevant results for the field, providing researchers with a normative set of data to explore. These included the degree of pervasiveness of false memories, incorrect information transmission, and illusory truth effects, among others (e.g., Gleaves et al., 2004; Fazio et al., 2015; see at this regard the classic Moses illusion paradigm; Song and Schwarz, 2008; Bottoms et al., 2010).

Tauber et al. (2013) asked different groups of college students from Kent State and Colorado State Universities to respond to the original questions in a computerized data collection. However, there were some constraints associated with the specificities of the data collection procedure (e.g., each participant completed only half the questions and a time limit was assigned to each experimental session). Due to these limitations, not all 671 participants completed all the questions, making the number of observations per question vary greatly (i.e., from 126 to 232 responses per question). These numbers are relatively lower than the original number of 270 responses per question collected by Nelson and Narens (1980). However, despite these differences, the results of a series of statistical tests examining the generational stability between the original and the updated norms demonstrated a high degree of steadiness across studies by means of rank-order correlations. More importantly, the results of item-level statistical tests between the recall in their pool as compared to that of Nelson and Narens's pool, also highlighted critical differences between the two sets, namely, a lower probability of recall, for nearly half of the questions (i.e., 139 questions out of the 299 that were tested). This clearly spoke to a shift in knowledge across the three-decade gap, and stressed the importance of the updated norms.

Stemming from the same line of reasoning, in the current study we provide the scientific community with an adapted version of the classic cultural norms commonly used in Anglophone countries (Nelson and Narens, 1980). Here we present the Spanish adaptation of general norms as a dataset of 132 of the original general-knowledge cultural questions recently validated and updated by Tauber et al. (2013). The ultimate aim of the current dataset is to provide the Spanish-speaking scientific community with a series of cultural questions related to different topics that could be used in a variety of studies in the field of psychology while following the general methods already used for the two normative studies run in the U.S. on these same items. The item and participant selection process will be detailed in the following sections and the general data-collection protocol will be described, followed by a comprehensive report of how data were treated and processed, as well as an overview of what the final datasets look like. The final dataset is stored in a public repository accessible to any researcher at: https://figshare.com/s/73927fadb0d35c7281db. The description of the files is presented below.

## Methods

### Participants

The final dataset comprises data from 294 native Spanish non-migrant participants. Spanish was the native language of all the participants. An initial sample that was somewhat larger was tested (317 participants), but a strict criterion was followed to retain only the data from those participants with an accuracy rate that demonstrated that sufficient attention had been paid to the task (set to a minimum of 20% of correct responses after visual inspection of the data). Hence, only data from 294 participants, who adequately completed the whole set of questions, were processed and analyzed (mean age = 21.14 years, SD = 3.29; number of females = 210). As seen, the number of the participants nearly matches the number reported by Nelson and Narens (1980) in their seminal study (i.e., 270 college students). Similar to the procedure followed by Nelson and Narens (1980), as well as by Tauber et al. (2013), participants were recruited from two different Spanish universities in order to favor replicability and generalization of the results. One hundred and seven participants were recruited from the Basque Center on Cognition, Brain and Language's (BCBL) pool of college students from the University of the Basque Country (UPV-EHU), and the remaining 187 participants corresponded to the student pool of the Universidad Europea del Atlántico (UNEATLÁNTICO).

### Materials

Even though the inclusion in the Spanish normative data collection of the whole set of 300 questions that Nelson and Narens (1980) initially tested might seem coherent, it should be kept in mind that in spite of the original authors' efforts, that pool of questions presents some limitations for cross-cultural norm collections, given that many of these questions are highly culture-dependent. For this reason, a two-step process was followed for the selection of final materials. First, all the questions that Tauber and colleagues marked as conflictive were discarded (see Table A3 from Tauber et al., 2013). Second, a subgroup of the remaining set of questions was selected with the intention of covering only cross-culturally valid knowledge that could also apply to Spanish college students (e.g., leaving out questions such as the last name of the first signer of the U.S. Declaration of Independence, or the name of the capital of Kentucky). Finally, and in order to make the data coding and processing feasible, only those questions with a non-synonymous, one-word expected correct answer were kept. This led to a final set of 132 questions that were translated to Spanish, and that are presented in Dataset-A and Dataset-B, as explained below.

### Procedure

The whole set of data was acquired during the first academic semester of 2016. All participants were tested by experienced researchers using PCs with the Experiment Builder software (SR-Research, Ontario, Canada). The 107 participants, who were tested at the BCBL, completed the experimental sessions individually in soundproof booths under the supervision of the researchers who monitored the activity from the outside. The 187 participants who completed the data collection in UNEATLÁNTICO, were tested in groups of approximately 25 persons at a time in a computer-equipped multimedia room in which each student was assigned a different PC. Except for this difference, the remaining process of data collection was identical across sites. Participants first provided a signed informed consent form and were briefly told about the aim of the experimental session and its basic procedural aspects. Participants were then assigned a PC and the instructions were displayed, indicating that they would be presented with a list of questions that they would have to rate for difficulty and then answer by entering their response using the keyboard. Participants were told that the questions varied greatly in the degree of difficulty, so that they would find easy questions as well as complicated ones. For each of the questions displayed, they were instructed to first report its degree of complexity or difficulty following a Likert-like scale from 1 (extremely easy) to 7 (extremely difficult). To do so, they were asked to press the numbers from 1 to 7 on the keyboard. Once this was done, they were instructed to respond to the question by typing the answer using the letters of the keyboard. They were explicitly told that if they did not know the answer they could guess, or they could enter the string “nls” (corresponding to “no lo sé,” Spanish for “I don't know”). They were also instructed to enter a single word as an answer, avoiding multi-word expressions or phrases. All the items were presented in a different random order to each participant, and several rest periods were interspaced with the trials to avoid fatigue. The whole experimental session lasted for approximately 75–90 min, depending on each participant's speed of response and typing skills.

## Data filtering and structure of the datasets

Much like the process followed by Tauber et al. (2013), once the complete set of data was collected, all answers were hand scored by the authors in order to adjust for spelling. Each individual response from the set of 38,804 answers to the questions^1^ was manually checked and the accuracy was determined after correcting the spelling mistakes. This way, all the words with unambiguous spelling errors were recoded in their orthographically correct form (e.g., from Dostoievsky to Dostoyevski). All these responses are presented in the raw data report Dataset-A, that includes the individual responses for each item given by each of the participants in a tab-delimited plain text format and in a Microsoft Excel® spreadsheet. While we acknowledge that the most relevant data compilation for researchers would be Dataset-B, which includes the averaged responses across participants for each of the 132 questions as explained below, we decided to present the raw data in Dataset-A to facilitate further analysis. Both final datasets constituting the Spanish General Knowledge Norms are accessible at https://figshare.com/s/73927fadb0d35c7281db as part of a public repository. Dataset-A contains a code for identification of each participant (PARTICIPANT), as well as a number from 1 to 132 that corresponds to each of the questions (QUESTION NUMBER), followed by the text of the question (QUESTION TEXT). Each question is also paired with the numbers that Tauber et al. (2013) reported for their questions according to the rank order (TAUBER QUESTION). Next, the answer provided by the participants is presented, followed by the answer that was expected in each case, that is, the correct answer (GIVEN ANSWER and CORRECT ANSWER, respectively). Dataset-A also indicates whether or not each response was correct (ACCURACY) by pairing each answer with one out of three possible characterizations: correct, incorrect, and unknown. All the items classified as unknown corresponded to the response “nls” (the string used by the participants to indicate that they did not know the answer and that they were not even able to guess it). Finally, the individual difficulty rate given by each participant to each question is also provided using the 1-to-7 Likert-like scale previously described (DIFFICULTY).

After individually checking and manually recoding all responses from the set, a series of indices were calculated for each of the 132 questions, constituting the core of the current data report. These pieces of information are reported in Dataset-B in the two same formats, that contain the data averaged across participants, and presents some of the basic identification tags also used in Dataset-A (QUESTION NUMBER, QUESTION TEXT, TAUBER QUESTION and CORRECT ANSWER), together with the critical indices of interest. For each item, we report the mean proportion of accuracy per participant (termed HIT RATE), the mean proportion of “nls” (I don't know) responses corresponding to a failure to retrieve an answer (called MISS RATE), and the mean proportion of incorrect responses (namely, the proportion of commission errors, called ERROR RATE). For each of the questions, the mean difficulty rate was also computed by averaging the difficulty scores according to the 1-to-7 Likert-like scale across participants (MEAN DIFFICULTY), provided along with the standard deviation (SD DIFFICULTY). Additionally, and following the same rationale of the analysis process used by Tauber et al. (2013), the most common commission errors were determined for each question, together with how often each of these commission errors occurred for that specific question (called MOST COMMON ERROR and MOST COMMON ERROR RATE, respectively). If the most common commission error corresponded to an idiosyncratic erroneous response given by a single participant, this specific error was not reported, leaving the field empty and setting the rate to 0. In the cases in which more than one different commission error occurred with the same frequency, all are reported (e.g., 7 participants thought that Holmes was the last name of the author who wrote the Sherlock Holmes stories, and another 7 thought that it was Shakespeare). Interestingly enough, some questions elicited a given commission error equally as often as the correct response. For instance, when asked for which country the yen is the monetary unit, 134 participants correctly identified that it was for Japan, while 133 thought that it was for China.

## Data overview and cross-cultural validation

**Figure 1:**
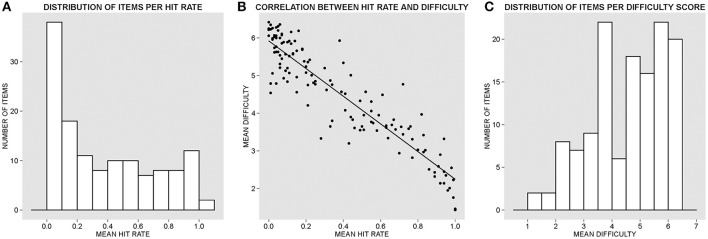
**(A)** Histogram of the distribution of items per hit rate (proportion of accuracy) in ascending order. **(B)** Correlation between hit rate and reported difficulty. **(C)** Histogram of the distribution of items per difficulty rate in ascending order according to the 7-point scale (1, extremely easy; 7, extremely difficult).

The present Data Report introduces the Spanish adaptation of the General Knowledge Norms first created by Nelson and Narens (1980) taken from a pool of U.S. college students who responded to 300 general-knowledge cultural questions. Tauber et al. (2013) updated and expanded the original norms in a more recent version used with different pools of U.S. college students. Following a procedure akin to that used in preceding studies and testing comparable samples obtained from Spanish-speaking college students, the current study provides the first cross-cultural normative validation of a database of general knowledge questions. Interestingly, the 132 questions that we report here represent a continuum of difficulty (see Figures 1A,C) useful in creating materials for future studies with Spanish stimuli aimed at exploring cognitive biases and effects that are modulated by prior knowledge (see Fazio et al., 2015, for an illustrative example with English stimuli taken from Tauber et al., 2013). As one would initially predict, the accuracy of the participants' responses was markedly correlated in a significantly negative manner with the reported difficulty (Spearman's ρ = −0.894, *p* < 0.001, *N* = 132), showing that participants' accuracy increased as an inverse function of the perceived difficulty of the questions (see Figure 1B). It is also worth noting that paired *t*-tests did not reveal significant differences between the mean accuracy scores obtained in the two different test sites, with a mean of 62.86% of errors (SD = 31.82) at the BCBL, and of 62.09% (SD = 33.49) at UNEATLÁNTICO [*t*_(131)_ = 1.21, *p* > 0.22). This suggests that the two subgroups of participants were relatively homogeneous in their cultural knowledge and provided similar responses. Furthermore, we ran a two-way random consistency interclass correlation test (see Shrout and Fleiss, 1979) in order to explore the level of agreement in participants' accuracy of response to each question, and results demonstrated an excellent degree of agreement among the average measures [ICC = 0.996, *F*_(130, 38090)_ = 262.52, *p* < 0.001].

An additional cross-cultural validation process was conducted in order to determine the degree of stability of these norms across cultures by taking Tauber et al.'s (2013) norms as a reference. A correlation analysis was carried out on the data from the whole set of questions between the hit rate observed in the current investigation and the hit rate reported by Tauber et al. for the English version of the same questions. The results showed that there was a significant positive correlation between the two proportions of accuracy (Spearman's ρ = 0.727, *p* < 0.001, *N* = 132). Interestingly, the correlation was not perfect, suggesting that in spite of the cross-cultural stability of part of the stimuli, some other items yielded a low degree of consistency across languages. Also, there was a significant negative correlation between the degree of confidence in the accuracy of response from the participants tested by Tauber et al. (2013), which is a proxy to the construct of ease of response, and the mean difficulty rates of the questions estimated by the Spanish participants (Spearman's ρ = −0.614, *p* < 0.001, *N* = 132). Hence, these results speak for the reliability of the current dataset by showing a high degree of consistency across the two cultures and languages tested so far (i.e., U,S, English and Peninsular Spanish), while also highlighting the usefulness and need for cross-cultural and cross-linguistic validations of general-knowledge question sets. We are confident that the current Data Report will give rise to a series of studies that will help expand and generalize the results obtained so far in English-speaking cultures.

## Ethics statement

The BCBL Ethics Committee approved the whole procedure and the methodological details prior to the data collection. All participants provided their informed signed consent prior to the data collection.

## Author contributions

JD developed the idea together with the help of LI. JD, LI, KG, and MS coordinated the data acquisition with the help of JM and MO. JD and LI analyzed the data. JD drafted the manuscript and all the authors approved the final version after discussing the intellectual content. All authors agreed to be accountable for all aspects of the work.

## Funding

This research has been partially funded by grants PSI2015-65689-P and SEV-2015-0490 from the Spanish Government, AThEME-613465 from the European Union and a personal fellowship given by the BBVA Foundation to the first author.

### Conflict of interest statement

The authors declare that the research was conducted in the absence of any commercial or financial relationships that could be construed as a potential conflict of interest.

## Footnotes

^1^This value corresponds to the whole set of responses that included the answers from 294 respondents to the 132 items, minus 4 answers to one specific question (QUESTION NUMBER = 1) that were lost due to an unexpected technical error.

## References

[B1] Bottoms H. C. Eslick A. N. Marsh E. J. (2010). Memory and the moses illusion: failures to detect contradictions with stored knowledge yield negative memorial consequences. Memory 18, 670–678. 10.1080/09658211.2010.501558 20706955

[B2] Fazio L. K. Brashier N. M. Payne B. K. Marsh E. J. (2015). Knowledge does not protect against illusory truth. J. Exp. Psychol. Gen. 144, 993–1002. 10.1037/xge0000098 26301795

[B3] Fazio L. K. Marsh E. J. (2008). Slowing presentation speed increases illusions of knowledge. Psychon. Bull. Rev. 15, 181–185. 10.3758/PBR.15.1.180 18605500

[B4] Gleaves D. H. Smith S. M. Butler L. D. Spiegel D. (2004). False and recovered memories in the laboratory and clinic: a review of experimental and clinical evidence. Clin. Psychol. Sci. Pract. 11, 3–28. 10.1093/clipsy.bph055

[B5] Marsh E. J. Balota D. A. Roediger H. L. III. (2005). Learning facts from fiction: effects of healthy aging and early-stage dementia of the Alzheimer type. Neuropsychology 19, 115–129. 10.1037/0894-4105.19.1.115 15656769

[B6] Marsh E. J. Meade M. L. Roediger H. L. III. (2003). Learning facts from fiction. J. Mem. Lang. 49, 519–536. 10.1016/S0749-596X(03)00092-5

[B7] Nelson T. O. Leonesio R. J. Shimamura A. P. Landwehr R. F. Narens L. (1982). Overlearning and the feeling of knowing. J. Exp. Psychol. Learn. Mem. Cogn. 8, 279–288. 10.1037/0278-7393.8.4.279 2939184

[B8] Nelson T. O. Narens L. (1980). Norms of 300 general-information questions: accuracy of recall, latency of recall, and feeling-of-knowing ratings. J. Verbal Learn. Verbal Behav. 19, 338–368. 10.1016/S0022-5371(80)90266-2

[B9] Shrout P. E. Fleiss J. L. (1979). Intraclass correlations: uses in assessing rater reliability. Psychol. Bull. 86, 420–428. 10.1037/0033-2909.86.2.420 18839484

[B10] Song H. Schwarz N. (2008). Fluency and the detection of misleading questions. Soc. Cogn. 26, 791–799. 10.1521/soco.2008.26.6.791

[B11] Tauber S. K. Dunlosky J. Rawson K. A. Rhodes M. G. Sitzman D. M. (2013). General knowledge norms: updated and expanded from the Nelson and Narens (1980) norms. Behav. Res. Methods 45, 1115–1143. 10.3758/s13428-012-0307-9 23344739

[B12] Weinstein Y. Roediger H. L. III. (2010). Retrospective bias in test performance: providing easy items at the beginning of a test makes students believe they did better on it. Mem. Cogn. 38, 366–376. 10.3758/MC.38.3.366 20234026

